# Nampt Expression Decreases Age-Related Senescence in Rat Bone Marrow Mesenchymal Stem Cells by Targeting Sirt1

**DOI:** 10.1371/journal.pone.0170930

**Published:** 2017-01-26

**Authors:** Cao Ma, Chenchen Pi, Yue Yang, Lin Lin, Yingai Shi, Yan Li, Yulin Li, Xu He

**Affiliations:** 1 The Key Laboratory of Pathobiology, Ministry of Education, College of Basic Medical Sciences, Jilin University, Changchun, China; 2 Department of Pathology, Zhongda Hospital, School of Medicine, Southeast University, Nanjing, China; 3 Center for Advanced Reconstruction of Extremities (C.A.R.E.), Sahlgrenska University Hospital, Mölndal, Sweden; Universita degli Studi di Udine, ITALY

## Abstract

Senescence restricts the development of applications involving mesenchymal stem cells (MSCs) in research fields, such as tissue engineering, and stem cell therapeutic strategies. Understanding the mechanisms underlying natural aging processes may contribute to the development of novel approaches to preventing age-related diseases or slowing individual aging processes. Nampt is a rate-limiting NAD biosynthetic enzyme that plays critical roles in energy metabolism, cell senescence and maintaining life spans. However, it remains unknown whether Nampt influences stem cell senescence. In this study, the function of Nampt was investigated using a rat model of natural aging. Our data show that Nampt expression was significantly lower in MSCs obtained from aged rats than in those obtained from young rats during physiological aging. Reducing the level of Nampt in aged MSCs resulted in lower intracellular concentrations of NAD+ and downregulated Sirt1 expression and activity. After the Nampt inhibitor FK866 was added, young MSCs were induced to become aged cells. The enhanced senescence was correlated with NAD+ depletion and Sirt1 activity attenuation. In addition, Nampt overexpression attenuated cell senescence in aged MSCs. Our findings provide a new explanation for the mechanisms underlying stem cell senescence and a novel target for delaying stem cell senescence and preventing and treating age-related diseases.

## Introduction

Cell senescence is a key characteristic of individual aging processes [1]. The aging of stem cells has been shown to be the cellular basis underlying many age-related diseases [2], such as Alzheimer’s disease, osteoporosis, and atherosclerosis [3]. However, age-related senescence limits the development of applications involving stem cells that can be used in tissue regenerative and cell therapeutic approaches. Based on our experience, the regenerative ability of mesenchymal stem cells (MSCs) that are obtained from aged individual is limited, and this severely restricts their therapeutic effects during autologous stem cell transplantation. Cell senescence is characterized by functional and morphological changes, such as irreversible growth cessation, metabolic abnormalities and fat brown pigment deposition [4,5]. In addition, aging cells display variations in senescence-associated-β-galactosidase (SA-β-gal) activity, oxidation levels, DNA damage, telomerase activity and the expression of senescence-associated factors [6–11]. In 2009, Imai proposed that “energy metabolism” might play a primary role in cell senescence. In mammalian cells, energy metabolism homeostasis is regulated by nicotinamide phosphoribosyl transferase (Nampt), nicotinamide adenine dinucleotide (NAD) and Sirt1 [12,13]. Nampt is the rate-limiting enzyme in the NAD re-salvaging pathway [14]. Hence, by influencing the synthesis of NAD, Nampt indirectly regulates the expression of Sirt1 [15]. Sirt1, a mammalian NAD-dependent protein deacetylase, subsequently deacetylates a large number of downstream signaling molecules that affect functional and morphological changes related to senescence [16].

Research on “NAD-related energy metabolism” has so far focused mainly on somatic cells. Our previous study revealed that the expression of Nampt was reduced in a time-dependent manner in MSCs undergoing replicative senescence *in vitro*. These data implied that Nampt may have regulatory effects on MSC senescence. We therefore hypothesized that Nampt may play a vital role in the regulation of natural senescence in MSCs in aged rats. In this study, we detected age-related changes in morphology, proliferation, and apoptosis to evaluate the biological characteristics of MSCs. Moreover, we measured senescence-related markers, including SA-β-gal activity, the cellular levels of reactive oxygen species (ROS), pl6^INK4A^ and p21^WAF1/CIP1^ expression, and markers of DNA damage and telomerase activity, during chronologic aging. In addition, to determine the regulatory function of Nampt during natural aging, we examined Nampt levels, Sirt1 expression and activity, and intracellular NAD+ concentrations in young and aged MSCs.

## Materials and Methods

### Ethics statement

All experimental protocols used in this study were approved by the Ethics Committee of Jilin University (Permit Number: SYXK 2013–0005).

### Animals and cell cultures

Male Sprague-Dawley (SD) rats were obtained from the Experimental Animal Center of Jilin University, Changchun, P.R. China. We used 1- to 2-month-old rats as the young group and 15- to 18-month-old rats as old group. Rats were sacrificed by cervical dislocation and primary MSCs were isolated from the bone marrows of rats in each group and cultured using the whole bone marrow adherent method. Briefly, MSCs were aseptically isolated from the femurs and tibias by using a 10 ml syringe to wash the marrow cavity. Then MSCs was harvested and plated into 10-cm culture dishes in complete medium containing 89% Dulbecco's Modified Eagle Medium with nutrient mixture F-12 (DMEM-F12, Gibco, USA) supplemented with 10% fetal bovine serum (Gibco, USA) and 1% penicillin streptomycin (HyClone, USA). The cells were incubated at 37°C with 5% CO_2_. After 24 hours of incubation, half of the complete medium was refreshed. The medium was then replaced every 3 days. When the cells became approximately 80% confluent, they were digested using 0.25% trypsin (HyClone, USA) and passaged at a 1:3 ratio. MSCs at passage 3 were used in subsequent experiments.

### Cell growth assay

In all, 5×10^3^ MSCs were seeded in each well of 24-well plates with complete medium. The cell numbers were counted in three wells per group for 7 days after cultivation.

### Cell doubling time

At the first time point (t1), we plated 7×10^5^ cells into each 10-cm dish, and this was designated the initial number of cells (Nf). Then, at time t2, we counted the number of cells (Ni) and determined the DT using the following formulas: PD = In(Nf/Ni)/In(2); Ct = t2-t1; DT = Ct/PD.

### Cell cycle analysis

The cell cycle analysis was performed using a Cell Cycle Detection Kit (KeyGEN BioTECH, China) according to the manufacturer’s instructions. Briefly, 1×10^6^ cells were collected and washed twice in PBS. The cells were then fixed in 70% methanol overnight at -4°C. The next day, the cells were centrifuged at 1500 rpm for 5 min, and the cell pellets were incubated with 100 μL of RNaseA for 30 min at 37°C. Then, 400 μl of propidium iodide (PI) was added to the cell suspension, and the cells were kept in the dark. The samples were then analyzed using flow cytometry.

### Senescence-associated β-galactosidase assays

To detect MSC senescence, senescence-associated-β-galactosidase (SA-β-gal) staining was performed using a senescence cell histochemical staining kit (Beyotime, China) according to the manufacturer’s instructions. Briefly, cells were fixed in fixation buffer for 15 min at room temperature. Cells were then washed with PBS and incubated in Staining Solution Mix overnight at 37°C. Cells were imaged under a bright-field microscope. The percentage of senescent cells was calculated by counting the number of blue cells (β-galactosidase-positive cells) out of at least 200 cells in different microscopic fields.

### Apoptosis evaluation

Cell apoptosis was determined by staining cells with a PI-AnnexinV Apoptosis Detection kit I (BD Biosciences, USA). A total of 1×10^6^ cells were collected and then washed twice with PBS. After the cells were centrifuged at 1500 rpm for 5 min, the cell pellets were resuspended in 1× binding buffer, and the cells were centrifuged again, as previously described, and the stained cells were incubated with 100 *μ*l of 1× binding buffer containing 5 μl AnnexinV/FITC and 10 *μ*L PI for 15 min at room temperature in the dark. Before the flow cytometry analysis, 400 *μ*L of 1× binding buffer was added to each sample.

### Intracellular ROS measurement

The intracellular accumulation of ROS was measured using a Reactive Oxygen Species Assay kit (Beyotime, China) according to the manufacturer’s instructions. A total of 1×10^6^ cells were collected and washed twice with PBS. Then, cells were then stimulated with medium containing 10 μM DCFH-DA for 20 min at 37°C. The cells were slightly shaken every 5 min. After removing the medium and washing the cells with serum-free culture medium, the cells were collected, and the fluorescence intensity of each sample was examined using flow cytometry.

### Comet assay

DNA damage was examined using a Comet Assay Kit (Trevigen, USA) according to the manufacturer’s instructions. A total of 4×10^3^ cells were harvested and mixed with low-melting agarose. The mixture was then spread evenly on a slide. After the mixture evolved from liquid to solid, it was placed in lysis buffer for 2 hours at 4°C. The slides were then immersed in alkaline unwinding solution (pH>13, 300 mM NaOH, and 1 mM EDTA) for 20 min at 4°C. The slides were submerged with the mixture in ice-cold electrophoresis buffer (pH>13, 300 mM NaOH, and 1 mM EDTA), and electrophoresis was then run at 300 mA for 20 min. The cells were quickly stained with PI and observed under a fluorescence microscope.

### Telomerase activity

Telomerase activity was analyzed using a TeloTAGGG Telomerase PCR ELISA Plus Kit (Roche, Germany) according to the manufacturer’s instructions. A total of 2×10^5^ cells were harvested and suspended in 200 μl of ice-cold lysis reagent. The cells were then incubated on ice for 30 min. The lysate was centrifuged at 12,000 rpm for 20 min at 4°C. The supernatant was carefully removed and transferred to a new tube for further TRAP assays. A solution containing 10 μg of protein extract was used for each assay. After amplification using PCR, the extended products were detected using ELISA.

### Gene expression measurement

Total RNA was extracted from MSCs using TRIzol (Takara, China), and reverse transcription was performed using a RNA PCR Kit (AMV)Ver.3.0 (Takara, China). Gene expression levels were determined using real time PCR with TransStart Top Green qPCR SuperMix (TRANS, China) in a 7300 Real-Time PCR System (ABI, USA). The primers were designed and synthesized as follows (Table 1). The data were normalized relative to the level of β-actin expression using the 2^−ΔΔCt^ method.

**Table 1:** Primers Used for qPCR.

PCR/ gene	Forward	Reverse
β-actin	GGAGATTACTGCCCTGGCTCCTA	GACTCATCGTACTCCTGCTTGCTG
pl6^INK4A^	AACACTTTCGGTCGTACCC	GTCCTCGCAGTTCGAATC
p21^WAF1/CIP1^	GACATCACCAGGATCGGACAT	GCAACGCTACTACGCAAGTAG
Nampt	AGGGGCATCTGCTCATTTGG	TGGTACTGTGCTCTGCCGCT
Sirt1	GCAGGTTGCAGGAATCCAAA	GGCAAGATGCTGTTGCAAAG

### Protein level measurement

Total protein was extracted using RIPA Lysis Buffer (Beyotime, China) according to the manufacturer’s instructions. Protein concentrations were determined using a BCA Protein Assay Kit (Beyotime, China). A total of 30 μg of each protein sample lysate was separated using 12% sodium dodecyl sulfate (SDS)-polyacrylamide gels and then transferred onto PVDF membranes. Nonspecific blots were inhibited by incubating the membranes with 5% nonfat milk in Tris-buffered saline (TBS) and the appropriate dilution of primary antibodies: anti-Nampt (diluted to 1:1000, BETHYL) and anti-Sirt1 (diluted to 1:1000, Santa Cruz) and overnight at 4°C. After the membranes were washed to remove excessive primary antibodies, the membranes were incubated for 1 hour at room temperature with secondary antibodies at an appropriate dilution of 1:2000. The membranes were washed three times and then visualized on X-ray film using Electro-Chemi-Luminescence (ECL) detection with ECL Plus (Beyotime, China). β-actin was used as an internal standard.

### Immunofluorescent staining

A total of 5×10^3^ cells were seeded into each well of a 24-well plate. When cells achieved nearly 50% confluence, they were fixed in 4% paraformaldehyde for 15 min at room temperature. Then, the cells were immersed in 0.1% Triton X-100. After inhibiting nonspecific binding with 1% bovine serum albumin at room temperature, we added 50 μL of anti-Nampt antibody (diluted 1:50, BETHYL), anti-Sirt1 (diluted 1:50, Santa Cruz) and incubated overnight at 4°C. On the next day, after the cells were washed three times with PBS, they were incubated for 1 hour with fluorescently labeled secondary AlexaFluor 594. Then, the samples were counterstained with Hoechst 33342 for 5 min, and the specific complexes were visualized under a fluorescence microscope.

#### Measurement of NAD amount

Intracellular NAD levels were detected using a NAD/NADH Quantitation Colorimetric Kit (BioVision, USA) according to the manufacturer’s instructions. Briefly, 2×10^5^ cells were suspended in 400 μl of NADH/NAD Extraction Buffer. The samples were frozen at -80°C for 20 min and thawed at room temperature for 10 min prior to quantitation. The cells were centrifuged at 12000 rpm for 5 min, and the NADH/NAD supernatant was transferred into a new tube. We heated the extracted samples at 60°C for 30 min and then directly transferred 50 μl of each sample into a well of a 96-well plate to detect NADH and NAD. We added a mixture of NAD Cycling Buffer and NAD Cycling Enzyme Mix to each well and then incubated the plate for 10 min at room temperature. The optical density of each well was then read at OD 450 nm. A standard curve was used to calculate the concentration of NAD+.

#### Measurement of Sirt1 activity

Sirt1 activity was measured using a SIRT1 assay kit (Sigma, USA) according to the manufacturer’s protocol. Briefly, 20 μl of protein was blended with 5 μl of NAD+ solution, and 10 μl of SIRT1 substrate solution was then added to the mixture, which was then incubated at room temperature for 10 min. Thereafter, 5 μl of developing solution was added, and the samples were incubated at 37°C for 10 min. Fluorescence was read using a plate reader, and the activity of the samples was calculated using a standard curve.

### Lentivirus transduction

The Rat Nampt coding region (NM_177928) was cloned into the pGC-FU-3FLAG-SV40-EGFP-IRES-puromycin lentivirus vector (GeneChem, China). The lentivirus vector and packaging plasmids expressing gag, pol, rev, and VSV-G genes (GeneChem) were transfectedinto 293T cells using transfection reagents (GeneChem, China). Viral supernatants were harvested at 48–72 hours after transfection and were concentrated by ultracentrifugation. A total of 1×10^4^ MSCs from old rats were seeded in each well of a 24-well plate, and cells were then transduced with lentivirus expressing Nampt in the presence of 5 μg/mL polybrene (Sigma-Aldrich, USA) for 10 hours. Seventy-two hours after infection, EGFP expression was monitored using a fluorescence microscope. Transduction efficacy was measured using Western blot and RT-qPCR.

### Statistical analysis

All data are expressed as the mean (±standard deviation). Comparisons between two groups were performed using a two-tailed Student’s t-test. p< 0.05 was considered statistically significant.

## Results

### Age-related changes in cell morphology

**Fig 1:**
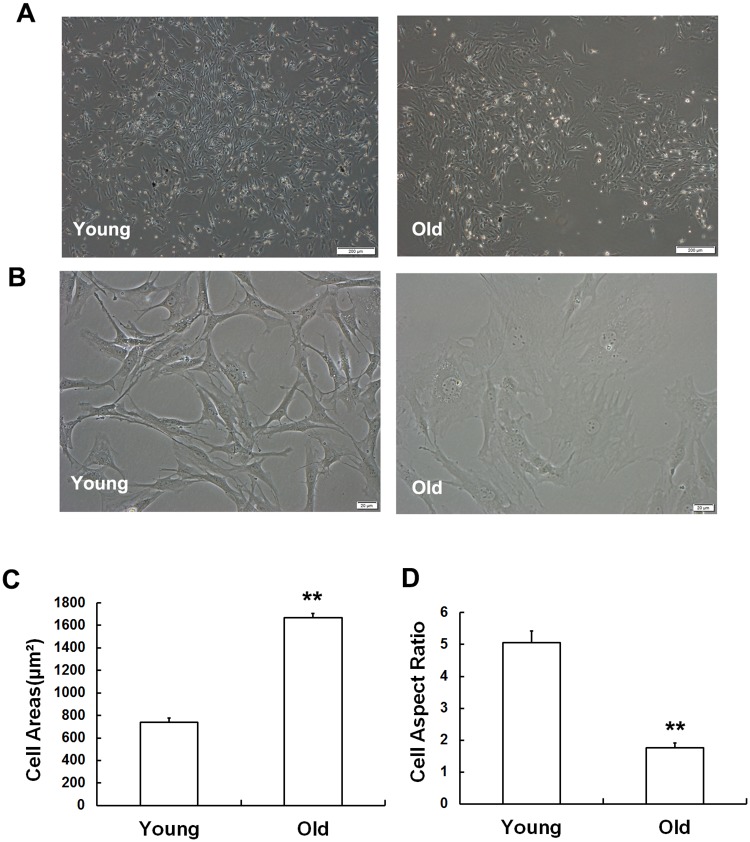
Age-related changes in cell morphology. Morphological changes were observed in MSCs using phase-contrast microscope. (A) No significant morphological differences were observed in primary MSCs between young and old rats. (B) MSCs at passage 3 in the young group maintained a long and fusiform shape, while cells in the old group appeared flattened and enlarged and had lost their stereoscopic perception. The cell areas were clearly larger (C) and the cell aspect ratios were markedly lower (D) in the old group than in the young group. Statistically analyzed values are shown and indicate the mean ± SD (*p< 0.05, **p< 0.01).

Cells were extracted using the whole bone marrow adherent method. The primary MSCs isolated from the young and old rats displayed similar morphological characteristics (Fig 1A). Cells at passage 3 in the old group appeared flattened and enlarged, lost their stereoscopic perception, and contained clearly visible particles in the cytoplasm, while cells in the young group grew normally. The cell borders between two adjacent cells were distinct, the cells were long and fusiform in shape, and they displayed a flocked arrangement (Fig 1B). The length and width of the cells were used to measure cell areas, and the results revealed that while the cell areas of the MSCs obtained from old rats progressively increased (Fig 1C), their cell aspect ratios gradually decreased (Fig 1D).

### Age-induced variations in proliferation and the cell cycle

**Fig 2:**
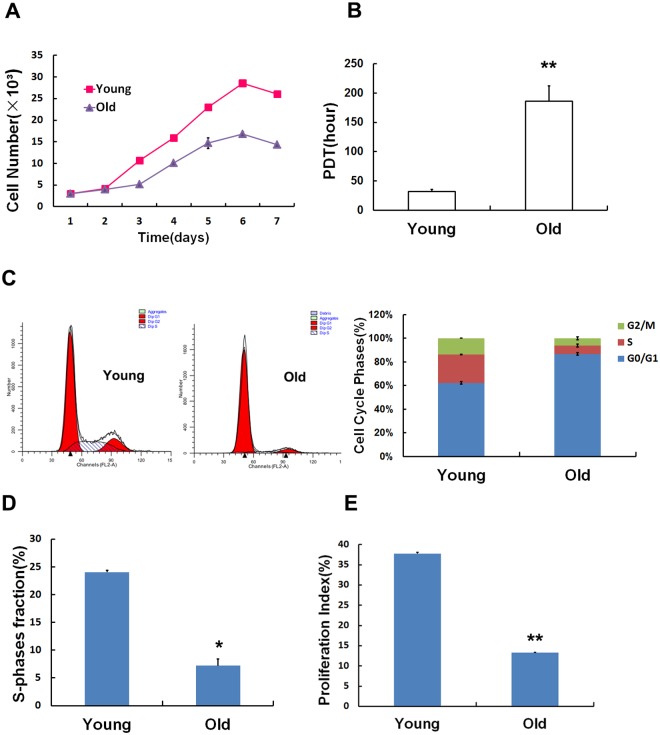
Age-induced variations in biological characteristics. Cell growth curves and population doubling times were used to detect changes in proliferation in both groups. In the MSCs obtained from the old rats, growth slowed (A), and population doubling times were extended (B). The cell cycle was analyzed using flow cytometry. Most of the MSCs in the old group were arrested in G0/G1 phase at the expense of S phase (C). The S-phase fraction (D) and proliferation index (E) were lower in the old group than in the young group. Values indicate the mean ± SD (*p< 0.05, **p< 0.01).

A statistically significant difference was observed in the cell growth curves of the MSCs between the 1- to 2-month-old rats and the 15- to 18-month-old rats (Fig 2A), indicating that MSCs isolated from old rats grow much slower. The population doubling time in the old group was markedly longer, by approximately 5-fold, than the doubling time in the young group (Fig 2B). In addition, the cell cycle also presented the following obvious distinction. MSCs from the young group displayed a prominent G1 peak and a lower proportion of S-phase cells (Fig 2C), while in the old group, G1 phase was longer, and the S period was shorter, suggesting that most of the cells were stuck in G1 phase, inducing permanent cell cycle arrest. Statistical analyses confirmed that the fraction of cells in S-phase (SPF) (Fig 2D) and the proliferation index (PI) (Fig 2E) were lower in the old group than in the young group.

### Cellular senescence increases in MSCs obtained from old rats

**Fig 3:**
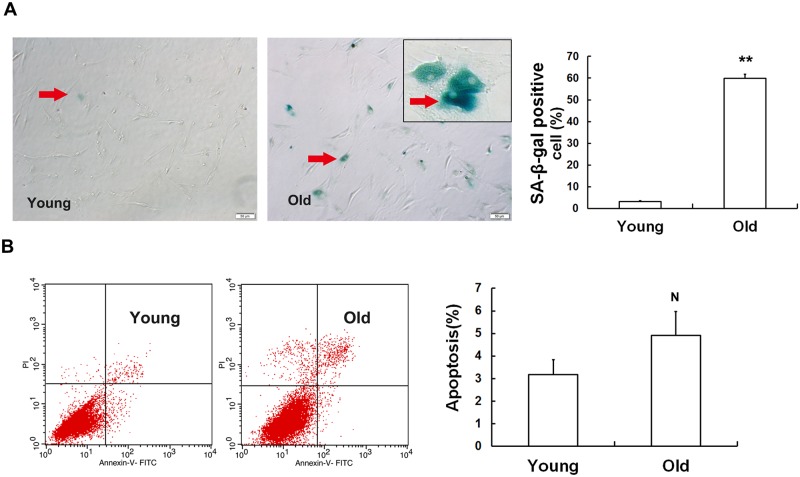
Cell senescence in MSCs obtained from old rats. (A) β-galactosidase staining. The ratio of SA-β-gal-positive cells was higher in MSCs obtained from old rats than in those obtained from young rats. As indicated by the arrows, senescent cells were stained a blue color. (B) Cell apoptosis was measured using flow cytometry. The results demonstrated that there was no significant difference in the rate of apoptosis between the young and old groups. The values shown indicate the mean ± SD (* p< 0.05, ** p< 0.01, ^**N**^ p> 0.05).

**Fig 4:**
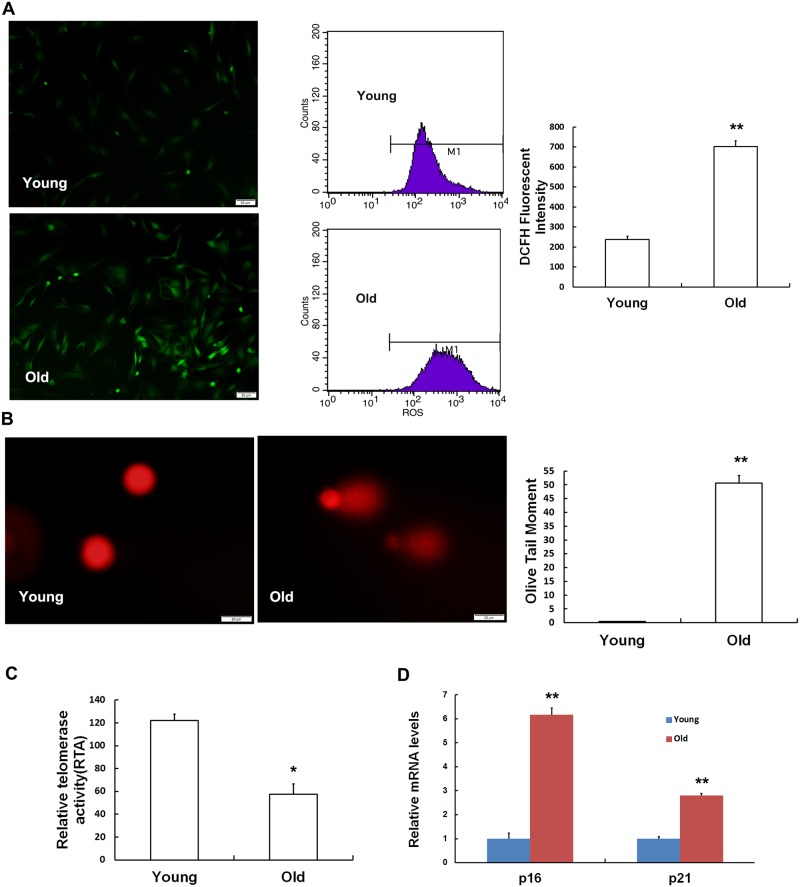
Cellular senescence is increased in MSCs obtained from old rats. (A) Intracellular ROS levels were determined using H2DCFDA staining and flow cytometry. The DCFH-fluorescent intensity was much higher in the old group than in the young group. (B) DNA damage was detected using comet assays. No clear comet tails were observed in the MSCs obtained from young rats, while almost every cell in the old group had a long and apparent comet tail. DNA damage was quantified by measuring olive tail moments (OTMs). The length of each OTM increased with the ages of the rats. (C) Telomerase activity was analyzed using a TeloTAGGG Telomerase PCR ELISA Plus Kit. Telomerase activity was clearly lower in the MSCs obtained from old rats than in the MSCs obtained from young rats. (D) Real-time qPCR analyses of the expression of the senescence-related genes pl6^INK4A^ and p21^WAF1/CIP1^. Actin was used as the reference gene. MSCs obtained from old rats expressed higher levels of the senescence markers pl6^INK4A^ and p21^WAF1/CIP1^ than were observed in the young group. The values shown indicate the mean ± SD (* p< 0.05, ** p< 0.01, ^**N**^ p> 0.05).

SA-β-Gal has been viewed as the gold standard for determining cell senescence [17]. The results of staining for SA-β-gal indicated that the ratio of SA-β-gal-positive cells was much higher in the MSCs obtained from old rats than those obtained from young rats (Fig 3A). However, a recent study revealed that SA-β-gal activity is also increased during apoptosis [18]. To eliminate the effect of apoptosis on our experimental results, we detected apoptosis in MSCs that were obtained from rats at different ages. Although the rate of apoptosis was higher in the old MSCs than that in the young MSCs, the difference between the two groups was not statistically significant (Fig 3B). To further confirm whether the number of senescent cells was increased in the old group, we used other methods to comprehensively judge cell senescence. As shown in Fig 4A, the intensity of ROS fluorescence was much weaker in the young group than in the old group, implying that MSCs in the old group generated more excessive ROS than the young group. Furthermore, the flow cytometry results were in accordance with the results of the fluorescence analysis results. Next, we performed a comet assay, and the quantitative results indicated that the length of OTM (Fig 4B) was higher in the old group than in the young group. We then tested telomerase activity and found that telomerase activity was clearly lower in the MSCs obtained from the older rats (Fig 4C). To evaluate cell senescence at the molecular level, we monitored the mRNA expression of classical senescence markers, including pl6^INK4A^ and p21^WAF1/CIP1^ using RT-qPCR (Fig 4D). We found that both pl6^INK4A^ and p21^WAF1/CIP1^ were expressed at different levels between the old group and the young group.

### Reduced expression of Nampt in chronologic aging

**Fig 5:**
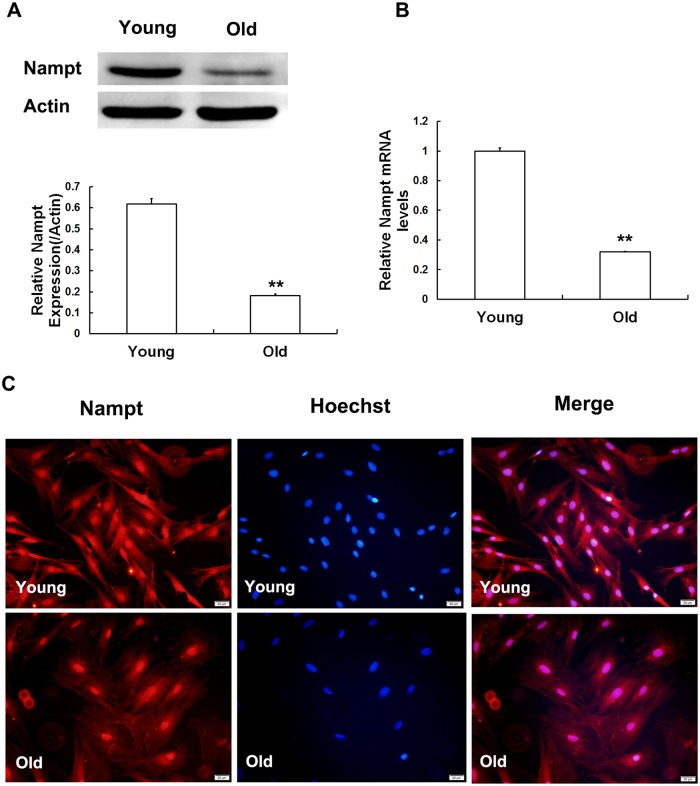
Nampt expression in MSCs obtained from young and old rats. Nampt protein levels were evaluated using Western blot analysis (A) and immunofluorescence (C). mRNA levels were detected using RT-qPCR (B). Nampt expression at both the protein and gene level were reduced in an age-dependent manner. Actin was used as the internal standard. The values shown indicate the mean ± SD (* p < 0.05, ** p< 0.01).

In our previous study, we found that Nampt expression decreased in a time-dependent manner in parallel with an increase in passages *in vitro*. To further explore whether there was a corresponding change in Nampt expression during chronologic aging, the expression of Nampt was examined in both groups. The protein (Fig 5A) and mRNA (Fig 5B) expression levels of Nampt were both lower in the MSCs obtained from the old group than in the young group by 3.12- and 3.39-fold, respectively. The immunofluorescence results were consistent with the results of Western blot analysis and RT q-PCR (Fig 5C). These data collectively indicate that the expression of Nampt was decreased in the MSCs obtained from the older group.

#### Lower Nampt levels in MSCs obtained from old rats were associated with lower levels of intracellular NAD+ synthesis, which attenuated Sirt1 expression and activity

**Fig 6:**
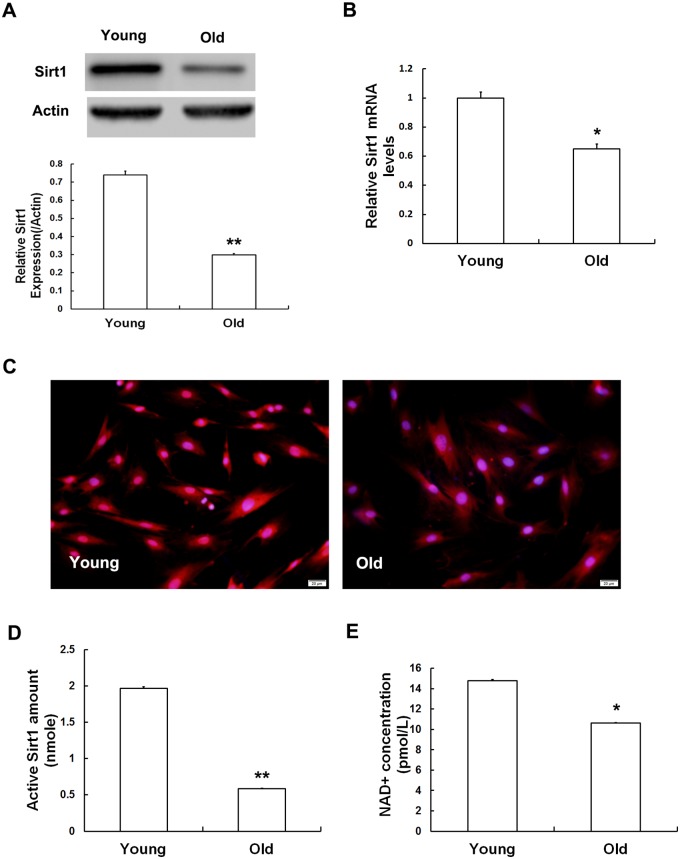
Sirt1 expression and activity and the intracellular levels of NAD+ in MSCs. Sirt1 protein levels were measured using Western blot analysis (A) and immunofluorescence staining (C), and mRNA levels were tested using real-time qPCR (B). Sirt1 activity was evaluated using SIRT1 Assay Kits (D). Intracellular NAD+ levels were detected using a NAD/NADH Quantitation Colorimetric Kit (E). Sirt1 expression and activity were dramatically lower in the old group than in the young group, and this decrease was associated with a reduction in intracellular NAD concentrations. The values shown indicate the mean ± SD (* p < 0.05, ** p< 0.01).

To further explore how Nampt depletion induces MSC senescence, we examined Sirt1 expression in MSCs obtained from both groups. The data showed that the Sirt1 protein expression was 2.34-fold lower (Fig 6A) and its mRNA expression was 1.54-fold lower (Fig 6B) than the levels in their young counterparts. Importantly, the results of immunofluorescence assays were consistent with the results of Western blot analysis and RT-qPCR (Fig 6C). In addition, we investigated Sirt1 activity in both MSC groups. As shown in Fig 6D, Sirt1 activity was 50% lower in old MSCs than in young MSCs. Furthermore, to determine how the downregulation of Nampt in old MSCs resulted in the attenuation of Sirt1 expression and activity, we evaluated intracellular NAD+ concentrations in both MSC groups. Our data showed that the MSCs derived from old rats displayed significantly lower intracellular NAD+ concentrations than were observed in the young rats (Fig 6E).

#### The Nampt inhibitor FK866 induced cell senescence

**Fig 7:**
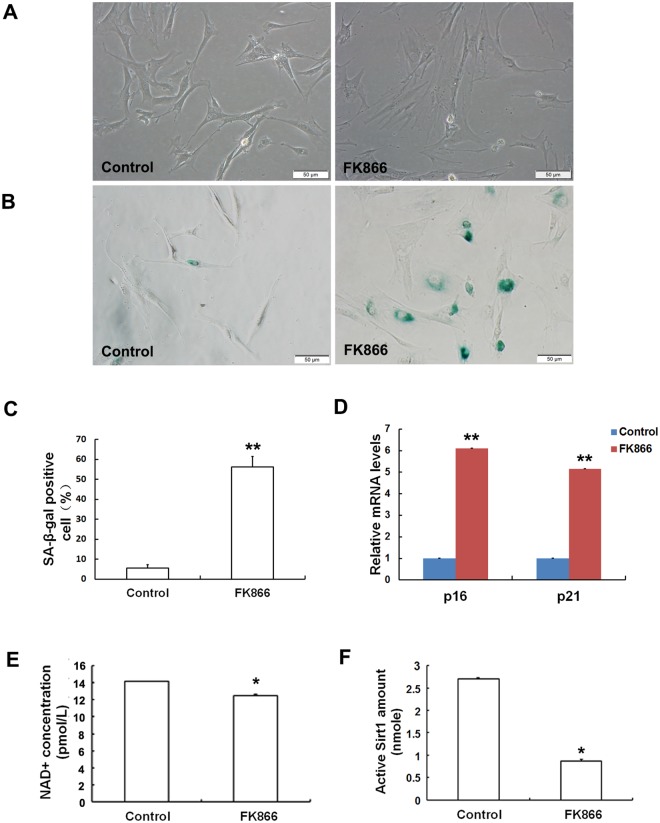
Inhibiting Nampt using FK866 increased cell senescence in MSCs obtained from the young group. Cells were treated with 10 nM FK866 for 72 h. (A) Unlike the controls, which displayed normal morphologies, the MSCs treated with FK866 were flattened and enlarged and displayed senescence-like morphological features. (B-C) SA-β-gal activity was then measured. The percentage of β-Gal-positive cells and their staining intensity were significantly higher in the FK866 group than in the control group. (D) pl6^INK4A^ and p21^WAF1/CIP1^ expression levels were evaluated using RT-qPCR. When Nampt was inhibited using FK866 in MSCs obtained from young rats, the expression levels of both pl6^INK4A^ and p21^WAF1/CIP1^ were upregulated. Furthermore, both intracellular NAD+ levels (E) and Sirt1 activity (F) were decreased in the FK866 treatment group compared to those in the control group. The values shown indicate the means ± SD (* p< 0.05, **p< 0.01).

To further confirm whether inhibiting Nampt induces cell senescence, we treated MSCs obtained from young rats with 10 nM FK866. After the addition of FK866, the cellular morphology of the MSCs was significantly altered (Fig 7A): the MSCs were flattened and enlarged, indicating that cellular senescence was induced by FK866 treatment. As shown in Fig 7B and 7C, SA-β-Gal activity was dramatically increased when cells were treated with 10 nM FK866. In addition, we also examined the expression levels of the senescence markers pl6^INK4A^ and p21^WAF1/CIP1^. The expression levels of both pl6^INK4A^ and p21^WAF1/CIP1^ were increased upon treatment with FK866. To further verify that FK866 induces MSC senescence via Sirt1 mediation, we then examined intracellular NAD+ concentrations and Sirt1 activity. As demonstrated in Fig 7E and 7F, NAD+ concentrations and Sirt1 activity in the FK866 treatment group were markedly lower than those in the control group. The results above indicated that FK866 could induce MSCs senescence, which was related to reduced NAD+ synthesis and decreased Sirt1 activity.

#### Nampt overexpression attenuated cell senescence

**Fig 8:**
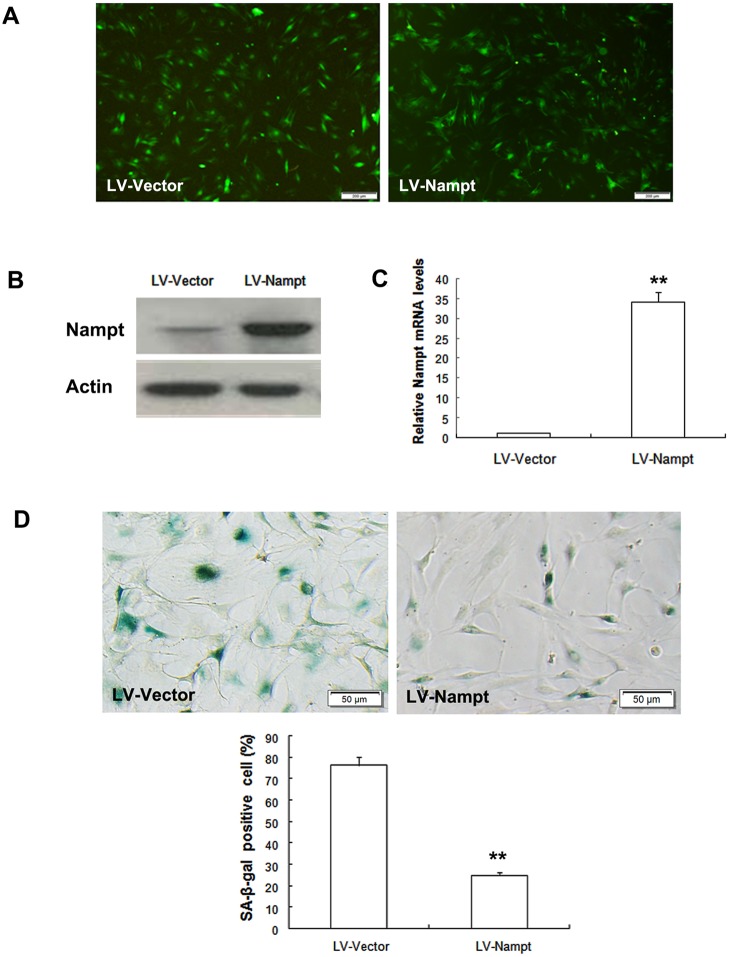
Nampt overexpression attenuated cell senescence in MSCs obtained from the old group. Cells were transduced with the lentivirus system. (A) Fluorescence images showed that Nampt was successfully overexpressed in MSCs from old rats. LV-Nampt: lentivirus encoding Nampt; LV-Vector: lentivirus encoding enhanced green fluorescent protein (EGFP). (B) Nampt protein levels were confirmed using Western blot analysis. (C) Nampt mRNA levels were ascertained using RT-qPCR. (D) Nampt overexpression significantly decreased SA-β-gal activity in MSCs from old rats.

To determine whether MSCs senescence could be weakened by enforcing Nampt expression, MSCs obtained from old rats were transduced with lentivirus expressing Nampt or the lentiviral vector. Fluorescence analysis revealed that Nampt was successfully overexpressed in aged MSCs (Fig 8A). Nampt expression was then confirmed at both the protein and mRNA levels by Western blot analysis (Fig 8B) and RT-qPCR (Fig 8C). To determine the effects of increasing Nampt expression on cell senescence, SA-β-Gal activity was further examined. We found that SA-β-gal activity in Nampt-overexpressed MSCs was obviously decreased compared to that in cells transduced with the vector (Fig 8D). The data here suggested that Nampt overexpression could attenuate MSC senescence.

## Discussion

With increasing age, the body inevitably experiences physiological or chronological aging, after which the organism is referred to as an aged or natural aging individual [19]. Cells extracted from aging individuals can be used to simulate the process of physiological aging. To minimize the impact of the external environment on cell aging, cells that were directly extracted from aging individuals were used as the research objects. In the present study, MSCs derived from both 1- to 2-month-old rats and 15- to 18-month-old rats were used to investigate the effects of age on biological characteristics and cellular senescence.

In our study, we found that there were clear differences in biological characteristics between old and young MSCs. Although primary MSCs were similar in morphology between the groups, at passage 3, the MSCs presented significant morphological differences. The MSCs obtained from the old group not only became enlarged and flattened but also lost their stereoscopic perception and displayed obvious cytoplasmic particles. The statistical data demonstrated that the cell aspect ratios were lower in the old group, while cell areas were increased. In addition, flow cytometry indicated that the number of MSCs that were arrested in the G1 phase, the S phase fraction (SPF) and the proliferation index (PI) were lower in the old group than in the young group. Along with the results shown in the cell growth curves, these data support the conclusion that the proliferation capacity of MSCs declines as a result of increasing age in rats. All evidence mentioned above indicates that MSC growth was permanently inhibited in the old group, suggesting that individual aging is accompanied by alterations in the biological features of MSCs.

To further confirm that senescence occurs in MSCs in old rats, we performed SA-β-gal staining [6,20]. The percentage of cells that were SA-β-gal-positive in the old group was approximately 60%, far higher than the percentage in the young group. Nevertheless, a recent study showed that enhanced SA-β-gal activity was also associated with apoptosis in lens epithelial cells [18], indicating that SA-β-gal-positivity is not completely representative of all aging cells. In our study, we found no significant difference in the rate of apoptosis between old and young MSCs. A growing amount of data indicates that ROS levels are elevated with the increasing age [21]. Increases in oxidative stress levels are another feature of senescent cells. We therefore investigated intracellular ROS levels using flow cytometry. We found that intracellular ROS levels were 3.5-higher in the old group than in the young group. Moreover, the degree of DNA damage also increased with age [22,23]. The comet assay can be used to estimate the extent of DNA breaks, which itself reflects the number of damaged cells and the severity of damaged [24]. In a sense, the longer the OTM, the more serious the DNA damage. This was confirmed by our findings, which showed that there were many more damaged cells in the old group than in the young group. In most cells, telomere length is maintained by telomerase activity [25]. When telomerase activity declines to a certain level, it becomes insufficient to overcome the progressive telomere shortening that occurs during replication and as a result of oxidative stress, and this results in senescence [26]. Sperka et al. proposed that a deficiency in telomerase activity was characteristic of aged stem cells [27]. In accordance with previous reports, the telomerase activity in the MSCs obtained from old rats was equivalent to only half of the activity observed in the young MSCs. Both pl6^INK4A^ and p21^WAF1/CIP1^ are cyclin-dependent kinase inhibitors that participate in the regulation of the cell cycle [10,28]. The downregulation of pl6^INK4A^ expression in mesenchymal stem cells derived from human dental pulp (DP-MSCs) clearly improved senescence-associated phenotypes during the physiological aging process [29]. Harris showed that pl6 ^INK4A^ and p21 ^WAF1/CIP1^ expression can be used as important biological indicators to determine whether adipose tissue-derived mesenchymal stem cells (AT-MSCs) senescence is or is not occurring [30]. These results indicate that MSCs isolated from old rats display senescence-associated phenotypes. In other words, MSC senescence naturally appears during physiological aging in individuals.

In 1957, it was reported that Nampt possesses enzymatic activity [31]. In further investigations, the roles played by Nampt in metabolism, aging, inflammation and other aspects have been gradually revealed. Especially interesting was the finding that Nampt may play an indispensable role in aging processes. Caloric restriction (CR) and exercise significantly upregulated Nampt expression, which contributes to prolonging the life expectancy of individuals and reducing the incidence of age-related diseases [32,33]. Nampt apparently extends the lifespan of human vascular smooth muscle cells by inhibiting the accumulation of p53 [34]. The relationship between Nampt and stem cell senescence has rarely been explored. In a previous study, we found that in MSCs, Nampt expression decreased in a time-dependent manner during the process of consecutive *in vitro* passages (not shown). However, it also remains unclear whether Nampt plays a similar role in natural senescence in MSCs in old rats. To explore this issue, Western blot analysis and real-time qPCR were used to detect the expression levels of Nampt. The results indicated that Nampt expression was dramatically lower at both mRNA and the protein level in the old group, which indicates that Nampt might play a regulatory role in natural aging in MSCs.

During the process of senile retinal degeneration, Sirt1 expression is significantly reduced [35]. Sirt1 can suppress the expression of pl6 ^INK4A^ and p21 ^WAF1/CIP^, reduce age-related DNA damage and enhance DNA repair abilities, thus postponing the onset of cellular senescence [36,37]. A recent theory proposed by Imai suggests that a Nampt/NAD+/Sirt1 cell expression profile constitutes “NAD world” and may represent a combination that modulates mammal aging processes [12–16]. Based on this theory, we hypothesized that Sirt1 expression and activity are downregulated in natural MSCs undergoing senescence and that this change is mediated by a reduction in the level of Nampt. To support this hypothesis, we evaluated the expression and activity of Sirt1. Our findings showed that Sirt1 expression and activity were both significantly lower in MSCs obtained from old rats than in those obtained from young rats. These results were supported by Chen and colleagues, who showed that the expression and activity of Sirt1 were much higher in MSCs in young rats than in MSCs in aged rats [38].

The NAD world theory states that the age-related downregulation of intracellular NAD levels is correlated with a decline in Nampt expression [13,33,39]. Based on this view, we speculated that intracellular NAD levels may be linked to reduced levels of Nampt and the downregulation of Sirt1 in senescent MSCs. This hypothesis was confirmed by our data, which shows that MSCs extracted from old rats contain clearly lower intracellular NAD+ concentrations than MSCs in young rats. Therefore, the NAD network might be involved in interactions between energy metabolism and cellular senescence in stem cells.

To confirm whether Nampt inhibition could lead to cell senescence, we treated young MSCs with the Nampt inhibitor FK866. As expected, after the addition of FK866, MSCs displayed a senescent morphology, increased SA-β-gal activity and elevated levels of senescent-related factors. The enhanced senescence was correlated with NAD+ depletion and Sirt1 activity attenuation. This finding is consistent with the recent report of Zhang and colleagues, who showed that NAD+ repletion improved stem cell function and enhanced life span in mice [40]. Recent studies demonstrated that Nampt overexpression extended the lifespan of smooth muscle cells and that Nampt was involved in the anti-aging process [34,41]. To strengthen our finding that Nampt inhibition induced cell senescence, MSCs from old rats were transduced with lentivirus expressing Nampt. Our results revealed that Nampt overexpression weakened cell senescence in aged MSCs. These findings indicate that Nampt plays a pivotal role in the regulation of MSC senescence.

Collectively, our results indicate that Nampt plays a regulatory role in natural senescence in MSCs. The mechanism underlying this role may involve reduced intracellular NAD concentrations, which are caused by declines in Nampt, which itself inhibits the expression and activity of Sirt1. Our findings provide not only an original explanation for the mechanisms underlying stem cell senescence but also a novel target for postponing stem cell senescence and developing potential preventive and therapeutic strategies for age-related diseases.
